# The conflict between oral health and patient autonomy in dentistry: a scoping review

**DOI:** 10.1186/s12910-024-01156-3

**Published:** 2024-12-21

**Authors:** Szilárd Dávid Kovács, Anggi Septia Irawan, Szilvia Zörgő, József Kovács

**Affiliations:** 1 Institute of Behavioural Sciences, Semmelweis University, Budapest, Hungary; 2 Maastricht University, Maastricht, the Netherlands

**Keywords:** Beneficence, Dental Ethics, Esthetics, Oral health, Personal Autonomy, Tooth extraction

## Abstract

**Background:**

Respect for patient autonomy, the principle that patients are capable to make informed decisions about medical interventions, is fundamental in present-day medicine. However, if a patient’s request is medically not indicated, the practitioner faces an ethical dilemma represented by the conflict of the principles of patient autonomy, beneficence, and maleficence. Adjacent to topics such as medical assistance in dying and healthy limb amputation, this ethical dilemma also manifests in the care of the maxillofacial region (the oral cavity and its surroundings), an area crucial to esthetic appearance, but also to everyday functions including mastication, speech, and facial expression, all of which are related to well-being. Our aim was to explore the manifestations and resolutions of the conflict between oral health and patient autonomy in relevant literature in order to contribute to the discourse of ethical challenges concerning patient autonomy, beneficence, and nonmaleficence.

**Methods:**

We screened all journal articles discussing the researched ethical dilemma obtained from three databases. Two researchers developed a hierarchical coding scheme, where the parent and grandparent codes were designated deductively as: *Case* (situations involving the researched ethical dilemma), *Judgement* (decisions made in the ethical dilemma), and *Principle* (ideas, rules, propositions explaining the judgements); child codes were developed inductively. After coding the sources, we utilized thematic analysis to construct code constellations.

**Results:**

Most themes identified in our sources advocated for the practitioner to choose the alternative that benefits the patient from a medical perspective, although no theme excluded the consideration of patient autonomy. Instances where respect for patient autonomy was encouraged concerned oral preventive care or when the requested intervention was expected to have an insignificant impact on oral health.

**Conclusions:**

Ethical conflicts concerning patient autonomy, beneficence, and nonmaleficence have a marked presence in oral care. These conflicts arise through the issue of body modification, evident in cosmetic dentistry and requests for tooth extraction. Our sources generally support the argumentation for beneficence, despite the rise of cosmetic procedures in dentistry.

## Background

Respecting patient autonomy, the principle that patients are capable to make informed decisions about medical interventions, stands as a foundational principle for decision-making in contemporary medicine [1]; nonetheless, literature often describes complexities beyond its simple, idealistic, and universally accepted application. The problem’s intricacy originates in the interaction of patient autonomy with other ethical obligations, most notably with the principles of nonmaleficence, beneficence, and justice, as elaborated by Beauchamp and Childress [1]. Scholars have created ethical models combining features of patient autonomy and beneficence. Bester distinguishes between an objective, biomedical aspect and a subjective, individual aspect of beneficence affiliated with the patient’s goals and values [2]. Similarly, Cohen’s non-discrete model claims that patient autonomy and beneficence determine each other, therefore the patient’s own request is the most beneficial for them, albeit the request must be medically sound to be regarded as such [3]. Models that consider beneficence paramount include Rubin’s collaborative model, in which patients desire and require the practitioner’s expertise in a process of shared decision-making [4]. Further arguments in favor of paternalism in certain situations claim that personal values are constantly evolving, therefore requests may only represent short term desires (as opposed stemming from a stable sense of self) [5]. Likewise, the model by Chen and Das describing physicians as “ontological decision architects” also promotes mild paternalism in favor of beneficence [6].

Despite the theoretical attempts to integrate patient autonomy and other ethical obligations, conflict between them emerges if a significant segment of the medical community disagrees with the procedure demanded by the patient. Illustrative examples provided by Goodman and Houk include healthy limb amputation and providing medical assistance in dying, asserting that granting patient autonomy in these cases is an unacceptable violation of the ethical principles of nonmaleficence and beneficence [7]. In contrast, others do not condemn medical assistance in dying, but voice concern for current trends and policies [8, 9].On the other hand, healthy limb amputation in Goodman’s and Houk’s view is a form of body modification, and likened to other practices such as cosmetic surgery, circumcision, and sex reassignment-surgery [7, 10, 11]. Despite the analogy in these cases, as the alleviation of negative emotions via body modification among patients with body integrity identity disorder seeking limb amputation and those undergoing sex-reassignment surgery, healthy limb amputation is generally less accepted [10].

Ethical challenges encompassing general well-being, bodily integrity, and patient autonomy are also pivotal in oral care due to the established link between oral health and quality of life [12]. Moreover, connections have been established between oral esthetics and multiple dimensions of life, such as career advancement, increased popularity, richer intimate experiences, elevated self-assurance, improved social abilities, and enhanced academic achievements [13–16]. Social emphasis placed on esthetic value is also evident in the increased public interest in cosmetic dental procedures, leading to a surge in demand for these services [17]. Surveys indicate that 13–38% of the general population has opted for vital tooth bleaching [18], and dental professionals report performing this procedure on a monthly basis [19].

Literature investigating the ethical challenges of oral health and patient autonomy in dentistry is sparse. Ozar, Sokol, and Patthoff propose a theoretical value hierarchy for the dental profession, ranking health above all other values, including patient autonomy [20]. Rule and Veatch contend a significant limitation of this hierarchical framework is the absence of consensus regarding the suggested prioritization [21]. Research synthesizing various sources has been conducted by Witter et al., whose literature review of wish-fulfilling medicine compiled examples of ethically challenging situations involving patient autonomy in dentistry [22]. Despite this advantage, in the discussion of the dental cases, they only referred to legal considerations.

Our objective in this study was to explore relevant literature regarding the manifestations and resolutions of the ethical conflict between patient autonomy and oral health in dentistry providing a comprehensive analysis of this lesser-explored area in medical ethics. Our research question was: in what cases is the conflict between oral health and patient autonomy present in literature, what judgements are made in these cases, and which principles guide these judgements.

## Methods

We obtained our sample with three individual searches conducted on the 28th of May 2023 in Scopus, Web of Science, and PubMed. Since our objective bridges medicine and social sciences, we utilized two databases encompassing a wide array of scientific fields (Scopus and Web of Science), while the third database focuses specifically on biomedicine and life sciences (PubMed) [23–26]. Our search terms were dent* AND ethic* AND autonomy AND health. All searches were refined to include articles written in English. We chose not to make the date of publication a sampling criterion, because despite evolving demands in dentistry, such as the growing importance of esthetics [17], focus on maintaining function and structure has remained consistently important over time. Search results in Scopus were further refined by setting the publication stage to *final*, and the source type to *journal*. Web of Science search results were further refined by setting the document type to *article* and *review article*. Duplicates were removed and screening for eligibility was carried out in two stages. In the first stage, we removed studies for which persistent access is not guaranteed by a DOI.In the second stage, two researchers screened the titles and abstracts for eligibility according to the following criteria: available abstract, discussing intervention targeting the oral cavity, discussing patient involvement in decision-making, and patients legally capable of providing informed consent (e.g., not minors). If screening the title and abstract was not conclusive, the researchers screened the full text of the article. The two researchers triangulated their screening results and resolved differences via social moderation.

In the next phase, we developed a hierarchical coding scheme. We established the parent codes (highest level of abstraction) deductively, based on Wide Reflective Equilibrium, a theoretical method aiming to achieve a coherent state among a set of conflicting beliefs [27]. Parent and grandparent codes were specified as follows: *Case* (situation where a dilemma occurs between oral health and patient autonomy), *Judgement* (decision made in a case) and *Principle* (ideas, rules, propositions explaining judgements). Parent codes also served as data segmentation; for this purpose, one researcher extracted article segments that fell within the scope of each parent code and placed them in separate text files, one containing all Cases, one for all Judgements, and one for all Principles.

Subsequently, two researchers developed child codes by performing free, inductive coding for each text file, employing the Interface for the Reproducible Open Coding Kit [28]. The two researchers triangulated their results and created a tentative codebook containing all parent and child codes. Both researchers test-coded the text files autonomously and proposed modifications to the tentative codebook. After a new round of triangulation, the two researchers repeated the test coding and approved the refined codebook for final coding, which was performed deductively by one researcher. The developed codebooks containing inductive code labels, definitions, and examples under parent and grandparent codes *Case*, *Judgement*, and *Principle* are displayed in Tables 1, 2 and 3, respectively.

**Table 1:** Codebook for the *Case* grandparent code

Grandparent code	Parent code	Child code	Code description	Example(s)
***Case*** A situation where the decision of an individual concerning a procedure targeting their oral cavity or surrounding tissues is described to be not (the most) beneficent for the integrity or function of the oral cavity	***Case nature*** The reason for or circumstances of the patient’s request	*Sociocultural*	Request for intervention due to adaptation of sociocultural norms (e.g., esthetic ideals, rituals, local identity)	Cosmetic preferences oppose bodily integrity; Nuer people’s local rituals involving tooth extraction
		*Personal experience*	Request based on personal reasons as opposed to sociocultural norms	Dental care phobia; Disorders of self-perception
		*Authority*	Intervention is proposed by the government, a practitioner, patient’s relatives, etc.	Artificial water fluoridation
		*Not specified 1*	The presence of the case with no details describing the circumstances or reasons	General descriptions of cases
	***Procedure*** The procedure (treatment or intervention alternative) in a *case*	*Extraction*	Patient request for tooth removal	Patient requests the removal of all their teeth
		*Prevention*	Intervention aiming to prevent oral disease from developing or aggravating. The aim is to prevent more invasive treatment.	Artificial water fluoridation; Oral hygienic care for geriatric patient
		*Not specified 2*	The presence of the case with no details of the procedure	General descriptions

**Table 2:** Codebook for the *Judgement* parent code

Parent code	Child code	Code description	Example(s)
***Judgement*** A decision that has been made in a *case*.	*Beneficial option*	Decision that does not align with the patient’s request.	A practitioner does not fulfil the patient’s request to remove all their teeth
	*Respecting autonomy*	Allowing individuals to choose a desired option and granting their request.	Claiming the caveat emptor principle; Arguing for patient autonomy
	*No definitive decision*	Postponing the decision.	Referring patient with an irrational request to psychiatric consultation; Persuading the patient to opt for a different treatment alternative

**Table 3:** Codebook for the *Principle* parent code

Parent code	Child code	Code description	Example(s)
***Principle*** Ideas, rules, propositions which explain the judgement(s)	*Professional imperative*	Recognizing the standards, customs or habits stemming from “the profession” or from academia	Describing not medically indicated decisions and “excuses”; Not considering non-scientific opinions; Comparing cosmetic dentistry to hair salons
	*Standard care*	Standards recognized by both the wider public and the profession, including the given sociocultural or legal environment	Legal reasons for a certain course of action; Avoiding action due to fear of legal backlash.
	*Impact*	The impact of an intervention is weighed to determine whether it is ethically acceptable	Emphasizing that tooth extraction is irreversible; Comparing oral epidemiology in areas with and without artificial water fluoridation; Advocating minimally invasive treatment
	*Patient needs*	References to any type of benefit for the patient; Emphasis to do no harm; Acts of paternalism	Unethicality of extracting teeth when medically not indicated; Claiming that the ethicality of an action is based on the diagnosis
	*Plurality*	Acknowledging alternative moral systems, ideals, habits; Including plurality of groups and individuals	Comparing ritual tooth extraction performed by the Nuer people to tooth extraction performed prior to orthodontic interventions in Western societies; Locality Rule in the USA; Claiming that if one dentist refuses extraction, a different dentist will grant the patient’s request
	*Individual decision-making*	Acknowledging individual freedom of choice, individual responsibility for health, or body identity	Emphasizing patient autonomy; Claiming tooth loss to be the individuals’ responsibility; Describing the oral cavity as an intimate area

To synthesize our results, we employed thematic analysis. One researcher revisited the dataset and identified code patterns across articles to draft themes within and across parent codes. Themes were refined by employing the constant comparison method for accuracy [29]. Subsequently, a researcher assigned labels to these themes and chose narratives that serve as exemplars [30–33]. Lastly, the research team as a whole validated the themes via social moderation. In the following, code and theme labels are indicated in italics.

## Results

### Literature search

**Fig. 1:**
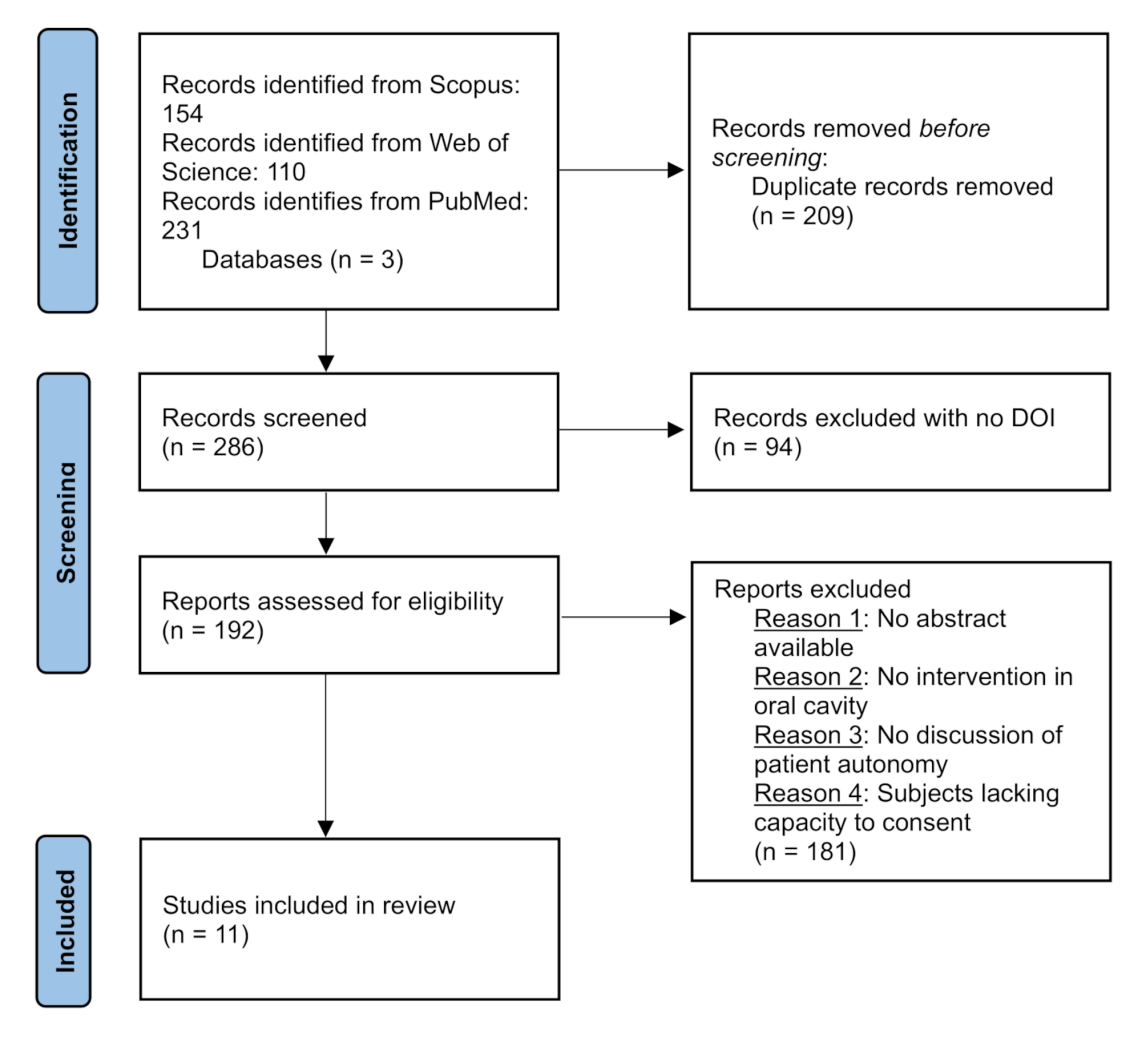
Preferred Reporting Items for Systematic reviews and Meta-Analyses (PRISMA) flowchart of study selection process

A total of 286 articles were screened and 11 were included. Figure 1 depicts the study selection process in a Preferred Reporting Items for Systematic reviews and Meta-Analyses (PRISMA) flowchart [34]. The specific exclusion criteria applied to records not included in the studyare available in our repository at: https://osf.io/aum29.

The included articles were published between 1988 and 2022, eight of which were published in dental journals, one in an agricultural journal, one in a public health journal, and one in a nursing journal. Six articles were theoretical in the sense that the authors did not work with empirical data; five articles conducted empirical research. Among the empirical studies, one explored the views of dentists in Australia [35], one in the Netherlands [36], and one did not specify the country in which the study was conducted [37]. Another empirical study reported the perspectives of non-dental healthcare practitioners in Sweden [38], and one empirical study reported patient beliefs in India [39]. The included articles and their relevant features are listed in Table 4.

**Table 4:** List of included articles and relevant features

	Author(s)	Journal	Year of publication	Study design	Objective(s)	Relevant ethical dilemma	Resolution of the ethical dilemma
Professional ethics and esthetic dentistry	Nash D.	The Journal of the American Dental Association	1988	Theoretical	No specified objectives	Patients’ esthetic ideals may compromise dentists’ professional standards	Carrying out interventions only in adherence to professional standards of care
Ethical issues encountered by dentists in the care of institutionalized elders	Bryant SR, MacEntee MI, Browne A.	Special Care in Dentistry	1995	Empirical (qualitative)	Explore ethical challenges dentists face when treating nursing home patients	Dentists reporting experiencing infringement in patient autonomy and dilemmas between nonmaleficence and patient autonomy	Some dentists adhere to an “autonomy model”, while other dentists adhere to a “beneficence model”
Paternalism, risk and patient choice	Baergen R, Baergen C.	The Journal of the American Dental Association	1997	Theoretical	No specified objectives	Patient with periodontitis requesting extraction of all teeth and fabrication of complete dentures due to sensitivity and dissatisfaction with appearance	No definitive resolution of the ethical dilemma
Ethical Dilemmas Confronting Dentists in Queensland, Australia	Porter SAT, Grey W.	Australian Dental Journal	2008	Empirical (quantitative)	Explore ethical challenges faced by dentists	Granting (unspecified) patient request if the dentist disagrees with the request	Majority of dentists believe the request should not be granted
Risk management in clinical practice. Part 5. Ethical considerations for dental enhancement procedures	Ahmad I.	British Dental Journal	2010	Theoretical	No specified objectives	Patients often request medically not indicated esthetic interventions	Patients should be able to make informed decisions about such interventions, however dentists may refuse to carry them out
Deciding about patients’ requests for extraction: ethical and legal guidelines	Broers DL, Brands WG, Welie JV, de Jongh A.	The Journal of the American Dental Association	2010	Theoretical	Provide ethical and legal guidelines for dentists	Various scenarios of patients requesting tooth extraction without medical indication	Focusing on the patient’s motivation without granting their request; No definitive answer for patients with body integrity identity disorder
Ethics of Artificial Water Fluoridation in Australia	Awofeso, N.	Public Health Ethics	2012	Theoretical	Answer ethical questions related to artificial water fluoridation	Artificial water fluoridation	The added beneficial value of artificial water fluoridation does not ethically justify the procedure
Ethical Approach to Fluoridation in Drinking Water Systems of UK and Turkey	Ateş, A., Özer, Ç.	Journal of Agricultural and Environmental Ethics	2017	Theoretical	Providing arguments for or against artificial water fluoridation	Artificial water fluoridation	Consuming fluoridated water should remain a personal choice
Healthcare providers’ experiences of assessing and performing oral care in older adults	Ek K, Browall M, Eriksson M, Eriksson I.	International Journal of Older People Nursing	2018	Empirical (qualitative)	Explore healthcare providers’ experience with maintaining oral health in geriatric patients	Geriatric patients often refuse aid with oral health routine	Coercion is not acceptable
Assessment of health-care ethical challenges in a dental hospital: A patient’s perspective	Chakrapani AR, Babitha GA, Prakash S, Prashanth G, Sushanth VH, Kumari N.	Journal of Indian Association of Public Health Dentistry	2021	Empirical (quantitative)	Evaluate patient opinions on various ethical challenges	Following prescribed (unspecified) treatment plans when in disagreement with the practitioner	Majority of patients deemed that following the prescribed treatment plan is necessary
Financial, psychological, or cultural reasons for extracting healthy or restorable teeth	Broers DLM, Dubois L, de Lange J, Welie JVM, Brands WG, Bruers JJM, de Jongh A.	The Journal of the American Dental Association	2022	Empirical (quantitative)	Determine the frequency of cases where patients request tooth extraction without medical indication, and how dentists manage such cases	Patient request for tooth extraction without medical indication	Majority of dentists granted the patients’ requests.

### Thematic analysis

#### Themes within grandparent code Case

##### Autonomous request for extraction

This theme was characterized by the co-occurrence of codes *Personal Experience* and *Extraction*. In these cases, patients seek tooth extraction despite their dentist’s recommendations, believing it to be the most beneficial for them. The reason cited by patients in all the sources in which the theme manifests was the fear of comprehensive dental treatment, whereas tooth extraction would immediately eliminate the patient’s oral symptoms [36, 40, 41]. Additionally, Broers et al. [41]. reviewed cases where patients requested tooth extraction to eliminate symptoms of mental disorders. In the case involving a patient suffering from somatoform pain disorder, the patient pursued comprehensive dental treatment before requesting tooth extraction as a final resort. This article also includes disorders of self-perception, e.g., body integrity identity disorder, where the dentition is not perceived as integral to their bodily identity [41]. 

##### Health promotion

This theme was marked by the co-occurrence of codes *Authority* and *Prevention*. A significant proportion of the cases we examined did not revolve around patient-initiated requests, but instead focused on cases where patients were subjected to preventive measures by authoritative figures. These cases manifested in two primary ways. Firstly, two articles discussed the involvement of the government as an authoritative entity mandating artificial water fluoridation to reduce the prevalence of dental caries [42, 43]. Authors argue that individual autonomy was compromised, as individuals did not have the choice of consuming non-fluoridated municipal water. Secondly, an ethical dilemma emerged within nursing homes on whether or not caregivers should enforce preventive oral hygienic practices on geriatric patients with inadequate oral care routines [38]. The dilemma in this case centered on the conflicting perspectives of patients’ relatives demanding maximal care, the nursing home staff striving for professional conduct, and the patients themselves refusing assistance with oral care.

#### Themes within parent code Judgement

##### Reviewing options

In this theme, the two seemingly opposing codes, *Beneficial option* and *Respecting autonomy*, co-occurred. Articles discussing both options, fulfilling, or rejecting the patient’s request, integrated the judgements of different individuals without arriving at normative conclusions, e.g., by reporting the results of a questionnaire. The respondents in the questionnaires were dentists [35] and patients [39]. Additionally, articles with a normative approach also argued that both options may be ethically viable [41, 44]. Overall, the articles in which this theme manifested, supported the notion that a single, overarching moral reality does not exist, aligning with the concept behind this study’s design, which incorporates a variety of moral viewpoints.

##### First, do no harm

In this theme, *Beneficial option* co-occurred with *No definitive decision*. Articles in which this theme manifested urged the practitioner to postpone the intervention demanded by the patient in hopes that the patient may finally opt for the beneficial alternative. Strategies for achieving this outcome included the elaboration of the risks of their desired treatment alternative, seeking consultation from other dentists, and referral to psychiatric consultation if the patient’s request was irrational. Nevertheless, if a definitive decision became inevitable, the sources advised practitioners not to grant the patient’s demand [36, 40]. 

#### Themes within parent code Principle

##### Shared decision-making

This theme was also marked by the co-occurrence of two seemingly opposing codes, *Patient needs* and *Individual decision-making*. Five reviewed papers imply the narrative that the primary objective of dental care is preserving the patient’s dentition as long as it provides adequate function. In pursuit of this objective, the sources advocated involving patients in a shared decision-making process and suggested the final decision to be made only if it was medically indicated [37, 40, 41, 43, 44]. Thus, this theme ranks beneficence over patient autonomy.

##### Limit for autonomy

This theme was represented by the co-occurrence of *Individual decision-making* and *Impact*. Five sources prioritized preserving the patient’s dentition [36, 40, 42–44], however, shared a less paternalistic approach than the previous theme. The sources argue medically non- indicated patient requests could be granted within a theoretical limit, which considers invasiveness, probability of complications, survival rate, and reversibility [44]. Hence, less invasive cosmetic procedures were permitted in this theme, although no specific examples were disclosed.

#### Themes across parent and grandparent codes

##### Professional ideals over society’s ideals

The constellation of codes *Sociocultural (Case)*,* Beneficial option (Judgement)*,* Patient needs (Principle)*, and *Individual decision-making (Principle)* constituted this theme. Two articles reviewed cases where patients requested tooth extraction in order to conform to societal norms. In a paper by Baergen and Baergen, a patient requested the removal of their symptomatic teeth and wished to receive an esthetic dental prosthesis [40]. Conversely, the article of Broers et al. discussed unique practices, where social norms did not align with western ideals of dental esthetics, for example, a ritual tooth extraction performed by the Nuer and Dinka peoples of Sudan [41]. This theme weighs patient autonomy, however overrules it, and advocates for the practitioners not to fulfil the patient’s demand [36, 40, 41, 44].

##### Autonomy in need for prevention

This theme involved the codes *Authority (Case)*,* Prevention (Case)*,* Respecting Autonomy (Judgement)* and *Individual decision-making (Principle)*, and correlated closely with cases discussed regarding *Health promotion*. All three sources in which *Autonomy in need for prevention* manifests agreed that preventive care should not be provided unless it is requested [38, 42, 43]. In the article discussing nursing home patients, the oral cavity was described as a private and intimate region, reinforcing the significance of the respect for patient autonomy and informed consent for interventions [38]. 

## Discussion

Recurring manifestations of the ethical dilemma between oral health and patient autonomy in dentistry are linked to cosmetic procedures, body modification, and the refusal of beneficial treatment. In this scoping review, we examined the cases, judgements, and principles in literature discussing the conflict between patient autonomy and oral health in dentistry.

The procedure that received the most attention in our sources was the removal of teeth, manifesting in the themes *Individual request for extraction* and *Professional ideals over society’s ideals.* Patients’ requests for tooth extraction were commonly linked to the immediate relief of pain, avoiding feared, exhaustive dental care, or an improved self-perception in patients suffering from body integrity identity disorder. Moreover, the request for tooth extraction may not only arise from perceived individual needs, but could also be influenced by social factors, associated with rituals observed in certain cultures. Previous studies investigating non-dental mutilation claim that the core of the ethical issue concerning such procedures is weighing postoperative dysfunction against psychosocial advantages [10]. However, our themes support the claim that professional standards associated with physical function are of the utmost importance and advised against carrying out bodily mutilation to prevent physical dysfunction including but not limited to speech, mastication, and facial expression.

A recurring element in our themes were judgements and principles indicating the primacy of the principle beneficence. Our results contradict practice, since cosmetic dental procedures are gaining popularity [18, 19], thereby possibly indicating a divide between theory and practice in dentistry. Furthermore, while non-dental body mutilations, such as healthy limb amputation and sex reassignment surgery, are paradoxically less commonly performed than dental cosmetic procedures or tooth extraction on request [18, 19, 45–48], the literature discussing the ethics of these procedures represents a wider range of perspectives.

While the majority of the examined sources advocated for the beneficial choice, the intervention’s impact was also highlighted when assessing its suitability, exemplified in the theme *Limit for autonomy*. This concept is supported by our observation that the only instance where patient autonomy was respected definitively was the refusal of preventive care, which does not immediately harm the patient (see theme *Autonomy in need for prevention*). Additionally, preventive care was the groups of interventions where the researched dilemma could directly manifest between the government and the populace, instead of the practitioner and patient. Recent ethical debates address the issue of serving the public by limiting individual autonomy in the case of mandatory vaccination [49, 50]. Our results confirm the arguments in favor of individual autonomy in a decision linked to relatively limited impact, as adequate oral care is possible without, for example, consuming fluoridated water [51].

In addition to respecting patients’ informed decisions, further considerations recognizing the patient’s personal identity were acknowledging their bodily integrity and the intimacy of the oral cavity, as well as entrusting the patient with the responsibility for their own health. Furthermore, patient autonomy was often viewed as a tool to gain consent for the medically indicated alternative. Thus, our sources tended to advocate for shared decision-making by integrating patient autonomy and beneficence. Unlike in Cohen’s non-discrete model, patient autonomy does not determine beneficence [3], rather remains a respected principle for providing beneficial treatment [4, 52].

The main limitation of this study is the low sample size due to the fact that the ethical dilemma in question has not been researched extensively. Additionally, most articles were published in dental journals, which may have a bias towards reporting practical aspects of interventions rather than capturing the nuanced experiences, values, and perspectives of patients. Furthermore, we did not weigh the moral validity, nor the soundness of the theoretical framework justifying the scholars’ claims; thus, our results are solely descriptive and explorative, and do not wish to resolve the ethical dilemma.

## Conclusions

Body modification, such as cosmetic procedures and tooth extraction are common practices in dentistry. Therefore, the ethical considerations of such interventions may offer parallels to other ethical dilemmas, where practitioners are obliged to choose between respecting the patient’s autonomy or serving the medically prescribed interest of the patient. The majority of our results supports the set of arguments that align with ranking beneficence and physical well-being first, and view autonomy as a tool to serve this purpose. Our study highlights a discrepancy between theory and practice due to the rise of cosmetic procedures in dentistry, in which patient autonomy serves to achieve psychosocial well-being.

## Data Availability

Our materials are available at: https://osf.io/xdbhm/.

## Declarations

### Authors’ information

SKD is a dentist and a PhD student. ASI is a medical anthropologist and a PhD student. SZ is an assistant professor in medical anthropology with expertise in qualitative data analysis. JK is a professor in bioethics, physician, and philosopher.
